# Supplementary material for: Modeling glioblastoma heterogeneity as a dynamic network of cell states
Source: Mol Syst Biol. 2021 Sep 16;17(9):e10105. doi: 10.15252/msb.202010105 (PMC8444284; doi:10.15252/msb.202010105)
Supplement: Supplementary file 6 — Source Data for Figure 5 [file MSB-17-e10105-s004.zip › Figure5A_sourcedata/GSEA_3071/nosigdb_stateVI.GseaPreranked.1622795169056/RICHARDS_ZHONG_NATURE_2018_OPC_UPREG.html]

Details for gene set RICHARDS\_ZHONG\_NATURE\_2018\_OPC\_UPREG[GSEA]

|  || Dataset | state63071 |
| Phenotype | NoPhenotypeAvailable |
| Upregulated in class | na\_neg |
| GeneSet | RICHARDS\_ZHONG\_NATURE\_2018\_OPC\_UPREG |
| Enrichment Score (ES) | -0.7207483 |
| Normalized Enrichment Score (NES) | -3.1064575 |
| Nominal p-value | 0.0 |
| FDR q-value | 0.0 |
| FWER p-Value | 0.0 |
Table: GSEA Results Summary

  

Fig 1: Enrichment plot: RICHARDS\_ZHONG\_NATURE\_2018\_OPC\_UPREG      
 Profile of the Running ES Score & Positions of GeneSet Members on the Rank Ordered List

  

| PROBE | GENE SYMBOL | GENE\_TITLE | RANK IN GENE LIST | RANK METRIC SCORE | RUNNING ES | CORE ENRICHMENT || 1 | ALCAM |  |  | 7 | 1.185 | 0.0203 | No |
| 2 | RHOC |  |  | 277 | 0.551 | -0.0443 | No |
| 3 | ARL4A |  |  | 704 | 0.395 | -0.1556 | No |
| 4 | EPB41L2 |  |  | 1343 | 0.299 | -0.3277 | No |
| 5 | OLIG2 |  |  | 1968 | -0.274 | -0.4965 | No |
| 6 | GATM |  |  | 2082 | -0.292 | -0.5225 | No |
| 7 | SERPINE2 |  |  | 2128 | -0.302 | -0.5293 | No |
| 8 | CNP |  |  | 2342 | -0.341 | -0.5823 | No |
| 9 | B2M |  |  | 2460 | -0.369 | -0.6079 | No |
| 10 | SLITRK2 |  |  | 2541 | -0.387 | -0.6230 | No |
| 11 | MMP2 |  |  | 2550 | -0.388 | -0.6179 | No |
| 12 | RAB31 |  |  | 2806 | -0.459 | -0.6803 | No |
| 13 | PLEKHB1 |  |  | 2810 | -0.461 | -0.6725 | No |
| 14 | TSPAN7 |  |  | 2848 | -0.475 | -0.6739 | No |
| 15 | PON2 |  |  | 2884 | -0.490 | -0.6745 | No |
| 16 | PDE4B |  |  | 2948 | -0.513 | -0.6824 | No |
| 17 | RFTN2 |  |  | 3065 | -0.570 | -0.7040 | No |
| 18 | OLIG1 |  |  | 3126 | -0.601 | -0.7095 | Yes |
| 19 | SCD5 |  |  | 3135 | -0.611 | -0.7002 | Yes |
| 20 | SPATA6 |  |  | 3143 | -0.614 | -0.6907 | Yes |
| 21 | ITGB8 |  |  | 3148 | -0.616 | -0.6802 | Yes |
| 22 | ITM2C |  |  | 3187 | -0.652 | -0.6786 | Yes |
| 23 | GNG7 |  |  | 3310 | -0.774 | -0.6980 | Yes |
| 24 | SPRY4 |  |  | 3325 | -0.793 | -0.6870 | Yes |
| 25 | DBI |  |  | 3349 | -0.812 | -0.6782 | Yes |
| 26 | SPARC |  |  | 3356 | -0.822 | -0.6644 | Yes |
| 27 | ETV1 |  |  | 3378 | -0.857 | -0.6542 | Yes |
| 28 | BCHE |  |  | 3405 | -0.909 | -0.6443 | Yes |
| 29 | CD82 |  |  | 3408 | -0.912 | -0.6278 | Yes |
| 30 | PLAT |  |  | 3416 | -0.927 | -0.6123 | Yes |
| 31 | SOX8 |  |  | 3418 | -0.934 | -0.5950 | Yes |
| 32 | CD9 |  |  | 3445 | -0.995 | -0.5836 | Yes |
| 33 | ETV5 |  |  | 3466 | -1.043 | -0.5696 | Yes |
| 34 | TTYH1 |  |  | 3481 | -1.067 | -0.5534 | Yes |
| 35 | TRIB2 |  |  | 3488 | -1.088 | -0.5347 | Yes |
| 36 | NOVA1 |  |  | 3510 | -1.148 | -0.5190 | Yes |
| 37 | DUSP6 |  |  | 3512 | -1.151 | -0.4977 | Yes |
| 38 | SOX6 |  |  | 3517 | -1.174 | -0.4767 | Yes |
| 39 | SEMA5A |  |  | 3526 | -1.210 | -0.4562 | Yes |
| 40 | LSAMP |  |  | 3537 | -1.257 | -0.4354 | Yes |
| 41 | LIMA1 |  |  | 3564 | -1.432 | -0.4158 | Yes |
| 42 | SPRY1 |  |  | 3565 | -1.432 | -0.3889 | Yes |
| 43 | SLC35F1 |  |  | 3576 | -1.554 | -0.3625 | Yes |
| 44 | SCRG1 |  |  | 3613 | -2.165 | -0.3319 | Yes |
| 45 | GPM6B |  |  | 3616 | -2.257 | -0.2900 | Yes |
| 46 | PMP2 |  |  | 3622 | -2.497 | -0.2445 | Yes |
| 47 | S100B |  |  | 3624 | -2.502 | -0.1978 | Yes |
| 48 | PTN |  |  | 3632 | -2.826 | -0.1467 | Yes |
| 49 | PDGFRA |  |  | 3636 | -3.635 | -0.0793 | Yes |
| 50 | PTPRZ1 |  |  | 3637 | -4.237 | 0.0003 | Yes |
Table: GSEA details [plain text format]

  

Fig 2: RICHARDS\_ZHONG\_NATURE\_2018\_OPC\_UPREG: Random ES distribution      
 Gene set null distribution of ES for **RICHARDS\_ZHONG\_NATURE\_2018\_OPC\_UPREG**
